# Supplementary material for: TUBB4A mutations result in both glial and neuronal degeneration in an H-ABC leukodystrophy mouse model
Source: eLife. 2020 May 28;9:e52986. doi: 10.7554/eLife.52986 (PMC7255805; doi:10.7554/eLife.52986)
Supplement: Figure 1—source data 1. [file elife-52986-fig1-data1.docx]

**Figure 1- Source data 1:**

**Data of mRNA expression in WT, *Tubb4a^D249N/+^* and *Tubb4a^D249N/D249N^* (Data provided as Mean**±**SEM)**

|  | **WT** | ***Tubb4a^D249N/+^*** | ***Tubb4a^D249N/D249N^*** |  | |
| --- | --- | --- | --- | --- | --- |
|  | **mRNA expression** | **mRNA expression** | **mRNA expression** | **p-value** |  |
| Cerebellum | 1.838 ± 0.17 | 1.839 ± 0.16 | 1.846 ± 0.25 | >0.99 |  |
| Cortex | 1.039 ± 0.26 | 1.141 ± 0.31 | 1.244 ± 0.41 | 0.85 |  |
| Hippocampus | 1.059 ± 0.23 | 1.088 ± 0.14 | 1.066 ± 0.51 | 0.99 |  |
| Hypo | 1.060 ± 0.18 | 1.236 ± 0.29 | 1.199 ± 0.25 | 0.85 |  |
| Prefrontal cortex | 1.127 ± 0.43 | 1.010 ± 0.33 | 0.925 ± 0.27 | 0.93 |  |
| Striatum | 1.366 ± 0.16 | 1.326 ± 0.09 | 1.522 ± 0.12 | 0.99 |  |
| Spinal cord | 1.530 ± 0.25 | 1.419 ± 0.15 | 1.420 ± 0.16 | 0.95 |  |
