# Supplementary material for: TUBB4A mutations result in both glial and neuronal degeneration in an H-ABC leukodystrophy mouse model
Source: eLife. 2020 May 28;9:e52986. doi: 10.7554/eLife.52986 (PMC7255805; doi:10.7554/eLife.52986)
Supplement: Figure 1—source data 2. [file elife-52986-fig1-data2.docx]

**Figure 1-Source data 2**

**Data of Behavioral tests (Data provided as Mean**±**SEM)**

| **Behavioral test** | **Age** | **WT** | ***Tubb4a^D249N/+^*** | ***Tubb4a^D249N/D249N^*** |
| --- | --- | --- | --- | --- |
| Ambulatory | P7 | 1.4 ± 0.16 | 1.4 ± 0.22 | 1.3 ± 0.15 |
|  | P10 | 2.2 ± 0.24 | 2.1 ± 0.27 | 1.2 ± 0.13 |
|  | P14 | 3 ± 0.00 | 3 ± 0.00 | 3 ± 0.00 |
| Ambulatory angle | P7 | 53.80 ± 3.58 | 53.10 ± 3.37 | 63.06 ± 4.04 |
|  | P14 | 43.16 ± 2.46 | 41.95 ± 2.98 | 71.82 ± 4.26 |
|  | P21 | 61.96 ± 2.93 | 64.51 ± 4.76 | 84.00 ± 7.56 |
|  | P28 | 43.20 ± 2.38 | 41.86 ± 2.61 | 80.88 ± 4.51 |
|  | P35 | 52.66 ± 1.87 | 44.11 ± 2.67 | 81.03 ± 5.37 |
| Grip fall angle | P14 | 103.9 ± 1.84 | 103.3 ± 2.68 | 86.4 ± 2.51 |
| Rota -rod | P21 | 217.8 ± 15.16 | 221.0 ± 13.85 | 107.1 ± 7.58 |
|  | P28 | 239.0 ± 10.76 | 242.72 ± 13.71 | 101.0 ± 10.30 |
|  | P35 | 257.9 ± 11.40 | 253.07 ± 4.76 | 20.69 ± 6.71 |
| Weights | P7 | 4.2 ± 0.27 | 3.7 ± 0.16 | 3.9 ± 0.22 |
|  | P14 | 7.3 ± 0.24 | 7.1 ± 0.18 | 6.8 ± 0.24 |
|  | P21 | 10.03 ± 0.30 | 9.8 ± 0.37 | 9.4 ± 0.31 |
|  | P28 | 14.21 ± 0.53 | 14.71 ± 0.37 | 13.56 ± 0.76 |
|  | P35 | 16.72 ± 0.48 | 17.64 ± 0.31 | 15.43 ± 0.76 |
|  | P36 | 17.12 ± 0.45 | 18.05 ± 0.34 | 15.31 ± 0.68 |
|  | P37 | 17.44 ± 0.43 | 18.57 ± 0.38 | 15.01 ± 0.67 |
|  | P38 | 17.93 ± 0.38 | 18.95 ± 0.40 | 14.66 ± 0.67 |
|  | P39 | 18.30 ± 0.34 | 19.34 ± 0.49 | 14.35 ± 0.74 |
|  | P40 | 18.71 ± 0.34 | 19.74 ± 0.57 | 14.03 ± 0.84 |
| Righting reflex | P39 | 0.00 ± 0.00 | 0.00 ± 0.00 | 4.30 ± 0.37 |
|  | P40 | 0.00 ± 0.00 | 0.00 ± 0.00 | 4.97 ± 0.66 |
