# Supplementary material for: TUBB4A mutations result in both glial and neuronal degeneration in an H-ABC leukodystrophy mouse model
Source: eLife. 2020 May 28;9:e52986. doi: 10.7554/eLife.52986 (PMC7255805; doi:10.7554/eLife.52986)
Supplement: Figure 2—source data 1. [file elife-52986-fig2-data1.docx]

**Figure 2-Source data 1:**

**Data of Myelin quantification (Data provided as Mean**±**SEM)**

| **Histology staining** | **Age** | **WT** | ***Tubb4a^D249N/+^*** | ***Tubb4a^D249N/D249N^*** |
| --- | --- | --- | --- | --- |
| Eri-C quantification | P14 | 0.35 ± 0.01 | 0.34 ± 0.04 | 0.16 ± 0.009 |
| (Corpus Callosum) | P21 | 0.79 ± 0.01 | 0.82 ± 0.04 | 0.18 ± 0.02 |
|  | End-stage (~P35-P40) | 0.95 ± 0.009 | 0.94 ± 0.009 | 0.05 ± 0.004 |
| Eri-C quantification | P14 | 0.65 ± 0.03 | 0.65 ± 0.07 | 0.30 ± 0.01 |
| (Cerebellum) | P21 | 0.79 ± 0.03 | 0.81 ± 0.03 | 0.27 ± 0.02 |
|  | End-stage (~P35-P40) | 0.85 ± 0.03 | 0.80 ± 0.02 | 0.03 ± 0.001 |
| PLP quantification (Corpus Callosum) | P14 | 63.76 ± 2.43 | 74.31 ± 11.5 | 54.16 ± 2.90 |
|  | P21 | 81.91 ± 8.65 | 80.80 ± 7.24 | 40.47 ± 2.51 |
|  | End-stage (~P35-P40) | 113.4 ± 16.1 | 124.75 ± 15.1 | 27.99 ± 3.02 |
| PLP | P14 | 28.74 ± 3.64 | 29.68 ± 4.06 | 24.98 ± 0.87 |
| quantification (Cerebellum) | P21 | 36.11 ± 4.75 | 38.85 ± 1.45 | 18.38 ± 1.54 |
|  | End-stage (~P35-P40) | 58.90 ± 8.19 | 48.54 ± 5.83 | 11.11 ± 1.17 |
| MBP quantification (Corpus Callosum) | P14 | 62.70 ± 3.80 | 61.76 ± 3.65 | 53.87 ± 4.56 |
|  | P21 | 74.30 ± 3.37 | 62.07 ± 6.54 | 29.20 ± 1.30 |
|  | End-stage (~P35-P40) | 94.07 ± 6.65 | 80.31 ± 5.90 | 25.73 ± 3.42 |
| MBP | P14 | 28.74 ± 3.64 | 26.08 ± 1.78 | 23.87 ± 0.44 |
| quantification (Cerebellum) | P21 | 41.61 ± 3.89 | 42.37 ± 2.20 | 18.87 ± 1.91 |
|  | End-stage (~P35-P40) | 69.79 ± 1.20 | 64.78 ± 2.58 | 12.65 ± 2.98 |
