# Supplementary material for: TUBB4A mutations result in both glial and neuronal degeneration in an H-ABC leukodystrophy mouse model
Source: eLife. 2020 May 28;9:e52986. doi: 10.7554/eLife.52986 (PMC7255805; doi:10.7554/eLife.52986)
Supplement: Figure 2—source data 2. [file elife-52986-fig2-data2.docx]

**Figure 2-Source data 2:**

**Normalized Western blots quantification of MBP and PLP (Data provided as Mean**±**SEM)**

| **Normalized values** | **Age** | **WT** | ***Tubb4a^D249N/+^*** | ***Tubb4a^D249N/D249N^*** |
| --- | --- | --- | --- | --- |
| PLP | P21 | 1.217 ± 0.36 | 0.956 ± 0.28 | 0.331 ± 0.07 |
| (Forebrain) | End-stage (~P35-P40) | 1.101 ± 0.01 | 0.68 ± 0.26 | 0.286 ± 0.08 |
| PLP (Cerebellum) | P21 | 0.876 ± 0.21 | 0.604 ± 0.29 | 0.069 ± 0.02 |
|  | End-stage (~P35-P40) | 0.860 ± 0.12 | 0.621 ± 0.23 | 0.123 ± 0.03 |
| MBP | P21 | 1.865 ± 0.28 | 1.794 ± 0.42 | 0.683 ± 0.10 |
| (Forebrain) | End-stage (~P35-P40) | 2.615 ± 0.09 | 2.188 ± 0.02 | 0.605 ± 0.06 |
| PLP (Cerebellum) | P21 | 2.279 ± 0.18 | 2.508 ± 0.24 | 1.647 ± 0.10 |
|  | End-stage (~P35-P40) | 2.306 ± 0.14 | 2.347 ± 0.12 | 1.307 ± 0.17 |
