# Supplementary material for: TUBB4A mutations result in both glial and neuronal degeneration in an H-ABC leukodystrophy mouse model
Source: eLife. 2020 May 28;9:e52986. doi: 10.7554/eLife.52986 (PMC7255805; doi:10.7554/eLife.52986)
Supplement: Figure 3—source data 1. [file elife-52986-fig3-data1.docx]

**Figure 3-Source data 1:**

**Counts/mm^2^ of Oligodendrocytes (OL) (ASPA), (OL precursor cells) OPC (NG2), OL lineage cells (Olig2) and Caspase in Corpus callosum ((Data provided as Mean**±**SEM)**

| **Type of cells** | **Age** | **WT** | ***Tubb4a^D249N/+^*** | ***Tubb4a^D249N/D249N^*** |
| --- | --- | --- | --- | --- |
| OL (ASPA+) | P14 | 313.9 ± 37.2 | 321.5 ± 26.8 | 158.1 ± 13.6 |
|  | P21 | 573.6 ± 29.3 | 577.6 ± 50.5 | 147.9 ± 8.38 |
|  | End-stage (~P35-P40) | 681.7 ± 38.4 | 720.8 ± 51.7 | 166.9 ± 32.34 |
| OPC (NG2+ Olig2+) | P14 | 1216.1 ± 49.1 | 1201.2 ± 32.6 | 1292.1 ± 103.0 |
|  | P21 | 1020.6 ± 29.9 | 1054.5 ± 61.4 | 1030.7 ± 28.6 |
|  | End-stage (~P35-P40) | 976.23 ± 45.4 | 1030.7 ± 63.3 | 1056.1 ± 42.5 |
| Olig2+ only | P14 | 2913.8 ± 102.2 | 2889.3 ± 56.70 | 2848.6 ± 169.8 |
|  | P21 | 2578.6 ± 74.49 | 2632.6 ± 127.4 | 2882.6 ± 108.3 |
|  | End-stage (~P35-P40) | 2516.0 ± 149.6 | 2800.3 ± 121.2 | 2785.3 ± 264.4 |
| Olig2+ Caspase+ | P14 | 4.861 ± 2.86 | 11.11 ± 7.85 | 38.99 ± 17.84 |
|  | P21 | 4.861 ± 2.86 | 11.11 ± 7.85 | 86.38 ± 24.26 |
|  | End-stage (~P35-P40) | 5.556 ± 3.20 | 4.762 ± 4.762 | 102.7 ± 18.21 |
