# Supplementary material for: TUBB4A mutations result in both glial and neuronal degeneration in an H-ABC leukodystrophy mouse model
Source: eLife. 2020 May 28;9:e52986. doi: 10.7554/eLife.52986 (PMC7255805; doi:10.7554/eLife.52986)
Supplement: Figure 4—source data 1. [file elife-52986-fig4-data1.docx]

**Figure 4-Source data 1:**

**Data of Electron microscopy (Data provided as Mean**±**SEM)**

| **Name** | **Age** | **WT** | ***Tubb4a^D249N/+^*** | ***Tubb4a^D249N/D249N^*** |
| --- | --- | --- | --- | --- |
| g-ratio for Optic nerve | End-stage (~P35-P40) | 0.81 ± 0.002 | 0.84 ± 0.003 | 0.92 ± 0.003 |
| g-ratio for Spinal cord | End-stage (~P35-P40) | 0.70 ± 0.004 | 0.80 ± 0.003 | 0.89 ± 0.003 |
| Normal % | End-stage (~P35-P40) | 82.51 ± 1.29 | 56.23 ± 3.81 | 5.807 ± 2.402 |
| Thin % | End-stage (~P35-P40) | 13.73 ± 2.155 | 39.07 ± 3.84 | 29.61 ± 1.60 |
| Unmyelinated % | End-stage (~P35-P40) | 3.75 ± 2.31 | 5.12 ± 2.02 | 64.57 ± 3.61 |
