# Supplementary material for: TUBB4A mutations result in both glial and neuronal degeneration in an H-ABC leukodystrophy mouse model
Source: eLife. 2020 May 28;9:e52986. doi: 10.7554/eLife.52986 (PMC7255805; doi:10.7554/eLife.52986)
Supplement: Figure 5—source data 1. [file elife-52986-fig5-data1.docx]

**Figure 5-Source data 1:**

**Counts/mm^2^ of Cerebellar granule neurons, striatal neurons and double positive Cerebellar granule+ caspase+ (Data provided as Mean**±**SEM)**

| **Neuron types** | **Age** | **WT** | ***Tubb4a^D249N/+^*** | ***Tubb4a^D249N/D249N^*** |
| --- | --- | --- | --- | --- |
| Cerebellar granular | P14 | 253.6 ± 6.93 | 243.9 ± 8.06 | 219. ± 13.6 |
|  | P21 | 312.7 ± 4.30 | 270.9 ± 7.86 | 212.7 ± 6.71 |
|  | End-stage (~P35-P40) | 262.8 ± 14.8 | 295.7 ± 10.4 | 57.52 ± 4.71 |
| Striatal neurons | P14 | 247.6 ± 9.61 | 238.4 ± 8.99 | 235.2 ± 6.35 |
|  | P21 | 231.3 ± 33.6 | 223. 8 ± 11.2 | 237.4 ± 8.15 |
|  | End-stage (~P35-P40) | 246.3 ± 28.5 | 218.8 ± 3.28 | 183.5 ± 8.44 |
| Cerebellar granular + Caspase + | P14 | 0.39 ± 0.16 | 0.47 ± 0.12 | 0.43 ± 0.06 |
|  | P21 | 0.66 ± 0.22 | 0.75 ± 0.38 | 11.41 ± 3.99 |
|  | End-stage (~P35-P40) | 0.75 ± 0.38 | 0.58 ± 0.22 | 13.50 ± 0.38 |
