# Supplementary material for: TUBB4A mutations result in both glial and neuronal degeneration in an H-ABC leukodystrophy mouse model
Source: eLife. 2020 May 28;9:e52986. doi: 10.7554/eLife.52986 (PMC7255805; doi:10.7554/eLife.52986)
Supplement: Figure 6—source data 1. [file elife-52986-fig6-data1.docx]

**Figure 6-Source data 1:**

**Data of OL culture and neuron culture (Data provided as Mean**±**SEM)**

| **Type of analysis** | **Parameter** | **WT** | ***Tubb4a^D249N/+^*** | ***Tubb4a^D249N/D249N^*** |
| --- | --- | --- | --- | --- |
| Total OL lineage | % of WT PLP/Olig2 counts | 99.66 ± 0.33 | 107.7 ± 12.9 | 117 ± 21.97 |
| Total mature OLS | % of WT PLP+ counts | 97.44 ± 3.69 | 72 ± 8.99 | 55.23 ± 4.97 |
| % of Mature OLs | % of WT PLP/Olig2 counts | 100 ± 0.57 | 58.8 ± 7.89 | 61.9 ± 5.31 |
| Neuron survival | % WT control | 100 ± 9.92 | 116.1 ± 6.08 | 69.53 ± 4.80 |
| Axonal outgrowth | Axon length | 176.4 ± 32.2 | 155.8 ± 24.4 | 118.4 ± 5.06 |
| Dendritic length | Dendritic length | 40.37 ± 5.66 | 38.49 ± 8.12 | 30.97 ± 2.46 |
