# Supplementary material for: TUBB4A mutations result in both glial and neuronal degeneration in an H-ABC leukodystrophy mouse model
Source: eLife. 2020 May 28;9:e52986. doi: 10.7554/eLife.52986 (PMC7255805; doi:10.7554/eLife.52986)
Supplement: Figure 7—source data 1. [file elife-52986-fig7-data1.docx]

**Figure 7-Source data 1:**

**Microtubule dynamics (Data provided as Mean**±**SEM)**

| **Type of analysis** | **WT** | ***Tubb4a^D249N/+^*** | ***Tubb4a^D249N/D249N^*** |
| --- | --- | --- | --- |
| Run time | 29.67 ± 0.79 | 28.55 ± 0.58 | 24.86 ± 0.59 |
| Run length | 7.208 ± 0.19 | 6.704 ± 0.14 | 6.003 ± 0.14 |
