# Supplementary material for: TUBB4A mutations result in both glial and neuronal degeneration in an H-ABC leukodystrophy mouse model
Source: eLife. 2020 May 28;9:e52986. doi: 10.7554/eLife.52986 (PMC7255805; doi:10.7554/eLife.52986)
Supplement: Transparent reporting form [file elife-52986-transrepform.docx]

***eLife’s* transparent reporting form**

We encourage authors to provide detailed information *within their submission* to facilitate the interpretation and replication of experiments. Authors can upload supporting documentation to indicate the use of appropriate reporting guidelines for health-related research (see [EQUATOR Network](http://www.equator-network.org/%20)), life science research (see the [BioSharing Information Resource](https://biosharing.org/)), or the [ARRIVE guidelines](http://www.plosbiology.org/article/info:doi/10.1371/journal.pbio.1000412) for reporting work involving animal research. Where applicable, authors should refer to any relevant reporting standards documents in this form.

If you have any questions, please consult our Journal Policies and/or contact us: [editorial@elifesciences.org](mailto:editorial@elifesciences.org).

**Sample-size estimation**

- You should state whether an appropriate sample size was computed when the study was being designed
- You should state the statistical method of sample size computation and any required assumptions
- If no explicit power analysis was used, you should describe how you decided what sample (replicate) size (number) to use

Empirical evidence based on published studies was used to determine n=10-20 animals per genotype for behavioral studies (PMC3826013, PMC3275906).

For histology and western blot analysis, we used n=3-4 animals per genotype. For cell culture studies, we had n=1-3 per genotype, with a technical replicate of n=3 and at least three independent studies were conducted. In addition, our H-ABC mouse model shows a robust phenotype justifying the numbers used above.

Please outline where this information can be found within the submission (e.g., sections or figure legends), or explain why this information doesn’t apply to your submission:

**Replicates**

- You should report how often each experiment was performed
- You should include a definition of biological versus technical replication
- The data obtained should be provided and sufficient information should be provided to indicate the number of independent biological and/or technical replicates
- If you encountered any outliers, you should describe how these were handled
- Criteria for exclusion/inclusion of data should be clearly stated
- High-throughput sequence data should be uploaded before submission, with a private link for reviewers provided (these are available from both GEO and ArrayExpress)

Please outline where this information can be found within the submission (e.g., sections or figure legends), or explain why this information doesn’t apply to your submission:

For all of the following experiments, “n” signifies the number of animals and is used as biological replicate in our analysis. We have technical replicates listed below which is sampling within each animal.

1. Behavioral studies: At least n = 10 (mentioned in supplemental methods and figure legends)

2. Histology: At least n=3 for each time point as biological replicate and also n=3-4 sections as technical replicate

3. Western blot: At least n=3 for each time point as biological replicate and technical replication was n=2.

4. Electron microscopy: At least n=3 for each time point as biological replicate and also n=3-4 sections as technical replicate

5. Cell Culture: At least n=1-3 biological replicate per genotype for each experiment and 3 independent experiments were conducted. For technical replicate we used at least and n=3 wells per animal

Samples and outliers were not excluded from the analysis.

**Statistical reporting**

- Statistical analysis methods should be described and justified
- Raw data should be presented in figures whenever informative to do so (typically when N per group is less than 10)
- For each experiment, you should identify the statistical tests used, exact values of N, definitions of center, methods of multiple test correction, and dispersion and precision measures (e.g., mean, median, SD, SEM, confidence intervals; and, for the major substantive results, a measure of effect size (e.g., Pearson's r, Cohen's d)
- Report exact p-values wherever possible alongside the summary statistics and 95% confidence intervals. These should be reported for all key questions and not only when the p-value is less than 0.05.

Please outline where this information can be found within the submission (e.g., sections or figure legends), or explain why this information doesn’t apply to your submission:

**Statistical analysis** – Statistical method used is described for each figure is mentioned in figures legend. All statistical analysis was performed using graph pad prism software and are described in method section.

**Reasoning** – Depending on the assay, if there are more than two groups and 2 different factors (genotype and age of mice), we chose two-way ANOVA. For example, we chose repeated measures two-way ANOVA, if we were comparing *different groups* (WT, Heterozygous mouse and Homozygous mouse) and across *different ages*. But if we had one factor, we chose one way ANOVA for example, Grip strength at P14.

Raw data or statistical values (SEM) are provided in Supplementary tables.

“n” is provided in figure legend.

Exact p – values are reported in figure legend and the Result section.

(For large datasets, or papers with a very large number of statistical tests, you may upload a single table file with tests, Ns, etc., with reference to sections in the manuscript.)

**Group allocation**

- Indicate how samples were allocated into experimental groups (in the case of clinical studies, please specify allocation to treatment method); if randomization was used, please also state if restricted randomization was applied
- Indicate if masking was used during group allocation, data collection and/or data analysis

Please outline where this information can be found within the submission (e.g., sections or figure legends), or explain why this information doesn’t apply to your submission:

We have not used randomization for our studies. However all of our studies were conducted in a blinded fashion, such as behavioral studies, image analysis, live cell imaging and tissue analysis.

**Additional data files (“source data”)**

- We encourage you to upload relevant additional data files, such as numerical data that are represented as a graph in a figure, or as a summary table
- Where provided, these should be in the most useful format, and they can be uploaded as “Source data” files linked to a main figure or table
- Include model definition files including the full list of parameters used
- Include code used for data analysis (e.g., R, MatLab)
- Avoid stating that data files are “available upon request”

Please indicate the figures or tables for which source data files have been provided:
